# An *in silico* platform for predicting, screening and designing of antihypertensive peptides

**DOI:** 10.1038/srep12512

**Published:** 2015-07-27

**Authors:** Ravi Kumar, Kumardeep Chaudhary, Jagat Singh Chauhan, Gandharva Nagpal, Rahul Kumar, Minakshi Sharma, Gajendra P.S. Raghava

**Affiliations:** 1 Bioinformatics Centre, CSIR-Institute of Microbial Technology, Chandigarh-160036, India

## Abstract

High blood pressure or hypertension is an affliction that threatens millions of lives worldwide. Peptides from natural origin have been shown recently to be highly effective in lowering blood pressure. In the present study, we have framed a platform for predicting and designing novel antihypertensive peptides. Due to a large variation found in the length of antihypertensive peptides, we divided these peptides into four categories (i) Tiny peptides, (ii) small peptides, (iii) medium peptides and (iv) large peptides. First, we developed SVM based regression models for tiny peptides using chemical descriptors and achieved maximum correlation of 0.701 and 0.543 for dipeptides and tripeptides, respectively. Second, classification models were developed for small peptides and achieved maximum accuracy of 76.67%, 72.04% and 77.39% for tetrapeptide, pentapeptide and hexapeptides, respectively. Third, we have developed a model for medium peptides using amino acid composition and achieved maximum accuracy of 82.61%. Finally, we have developed a model for large peptides using amino acid composition and achieved maximum accuracy of 84.21%. Based on the above study, a web-based platform has been developed for locating antihypertensive peptides in a protein, screening of peptides and designing of antihypertensive peptides.

In the past, various types of bioactive peptides have been discovered, these peptides play vital role in various types of activities *e.g.* opioid^1^, antihypertensive^2^, cell penetrating^3^, tumor homing^4^, antimicrobial^5^, anticancer^6^, hemolytic peptides^7^, antiparasitic peptides^8^, dipeptidyl peptidase inhibiting, anti-amnesic, antithrombotic, etc. Numerous bioactive peptides have been reported from food proteins that can be obtained by fermentation or enzymatic hydrolysis of these proteins. The bioactive food peptides having antihypertensive activities are receiving attention due to their role in cardiovascular diseases, which is a major cause of deaths worldwide^9^. There are a number of methods used for producing antihypertensive peptides (AHTPs) including enzymatic hydrolysis, fermentation and use of recombinant bacteria^10^.

There are a number of medicines commonly used to treat hypertension like nitrates, beta-blockers, diuretics, vasodilators, dopamine agonist calcium channel blockers^11^. Though most of the existing antihypertensive drugs are highly effective but they have side effects *e.g.* skin rashes, hypotension, dry cough, increased potassium level, taste disturbance^12^. Thus, it is always challenging to discover or design safer drugs for prevention and treatment of hypertension. In past, attempts have been made to extract antihypertensive compounds from natural resources *e.g.* wheat, potato, etc. It was observed that certain food products (*e.g.,* milk, egg, soy, muscle proteins, pea, garlic, rice, etc.) contain antihypertensive peptides^10^,^13^. In addition, several animal sources have been reported to contain antihypertensive peptides *e.g.* muscle, ovalbumin, blood, fish protein, pork meat etc.^14^,^15^. Before AHTPDB^16^, only limited resources were available on antihypertensive peptides e.g. ACEpepDB, which contains about 865 peptides from different sources (http://www.cftri.com/pepdb/). In addition, few quantitative structure activity relationship (QSAR) based regression models have been developed for predicting inhibitory activity for tiny peptides^17^,^18^. There is no prediction method available for small, medium and large peptides. In our recent work, we have compiled antihypertensive peptides from various resources and built a database of antihypertensive peptides, AHTPDB^16^.

In this study, a systematic attempt has been made to develop models for predicting antihypertensive (AHT) peptides. It was observed that the length of antihypertensive peptides has a large variation. Thus, in this study, we developed four types of models for predicting AHT peptides of various sizes. We used machine-learning techniques for developing prediction models. One of the novelties of this study is web-based platform, AHTpin, developed for designing AHT peptides. AHTpin is a user-friendly platform providing various options to the users for predicting, designing and screening of AHT peptides. It is freely available at the URL: http://crdd.osdd.net/raghava/ahtpin.

## Methods

### Datasets

**Figure 1:**
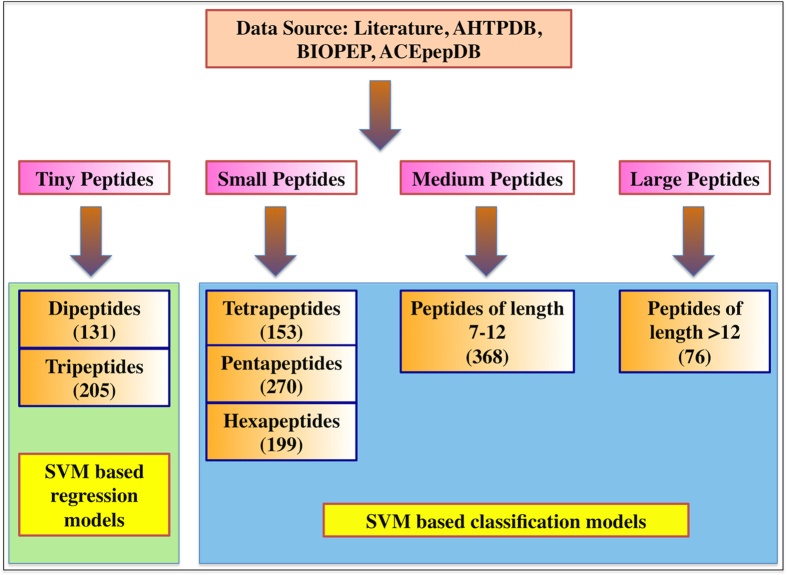
Schematic diagram showing the datasets used for the development of different models.

We extracted 1745 antihypertensive peptides (AHTPs) from literature and publically available databases like AHTPDB^16^, BIOPEP^19^ and ACEpepDB (http://www.cftri.com/pepdb). We excluded the peptides having non-natural amino acids. Based on the length of peptides we created four types of datasets. We developed regression and classification models as shown in Fig. 1. Following is the brief description of datasets used in this paper:

### Tiny peptides

We assigned dipeptides and tripeptides in the category of tiny peptides. Our datasets contain 131 dipeptides having inhibitory activity (IC_50_) between 0.92 to 17000 μM; 205 tripeptides having IC_50_ between 0.04 to 2700 μM. We developed regression models to predict inhibitory activity of these peptides. We have converted IC_50_ value into normalized pIC_50_ values {=−log (IC_50_ × 10^−6^)} to narrow down the scale. In order to perform external validation of our models that is in compliance with OECD principles^20^,^21^,^22^,^23^,^24^,^25^,^26^, we created independent datasets (20 dipeptides and 40 tripeptides). These peptides were selected randomly from the main datasets with satisfying the condition of two-column statistics. Rest of the dipeptides (111) and tripeptides (165) were used as training datasets to develop models for external validation.

### Small peptides

The peptides with number of residues, four, five or six have been classified into small peptides. We obtained total 153 tetrapeptides, 270 pentapeptides and 199 hexapeptides having antihypertensive activity as positive examples. Classification based prediction models have been developed for small peptides.

### Medium peptides

All peptides having number of residues between 7 and 12 (inclusive) are called medium peptides in this study. We developed single classification model for these peptides. This medium peptide dataset contains 368 AHTPs.

### Large peptides

There are few AHTPs having number of residues more than 12; we categorized these peptides as large peptides. We developed classification models for these peptides. Our large peptides’ dataset contains 76 AHTPs.

### Negative Dataset

In the absence of experimentally validated non-antihypertensive peptides (non-AHTPs), we obtained random fragments of the same length from the Swiss-Prot proteins and used them as negative datasets. For instance, in case of tetrapeptides, equal number of random tetrapeptides were taken from the protein sequences in Swiss-Prot and used to constitute the negative dataset (provided they were not present in the positive dataset). This method of taking random sequences as negative dataset is a routinely used standard procedure^27^,^28^,^29^,^30^ and it is based on the assumption that probability of finding the random sequences to be positive is very low.

### Prediction features

In this study, we have used three kinds of peptide features for developing models. These are amino acid composition, atomic composition and chemical descriptors; following is the brief description of these features:

#### (i) Amino acid composition

Amino acid composition in proteins/peptides is well conserved from species to species and different class of proteins/peptides, so amino acid composition can be distinguishing feature to discriminate two classes of protein/peptides^31^. Amino acid composition represents the fraction of each amino acid in a peptide, and it is represented by the vector of 20, corresponding to each of the 20 amino acids. Amino acid composition was calculated using the following equation (equation 1):


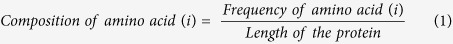


Where *i* can be any natural amino acid.

#### (ii) Atomic composition

In this composition, we have calculated frequency of each atom in amino acid. Natural amino acids are made up of five types of atoms (C, H, N, O, S), thus first we used these five atom frequencies as prediction features. As peptides are made up of 20 types of amino acids, so we computed number and type of atoms in each amino as given in Table 1.

#### (iii) Chemical descriptors

Chemical descriptors play an important role in determining the biological activity of any chemical molecule. Therefore, they were used as critical features in developing QSAR models in the past. In this study, we used open source software PaDEL for calculating different types of descriptors^32^. We used this software for calculating 15,537 types of descriptors, including 1D, 2D, 3D and 10 different types of binary fingerprints (further information about these descriptors is available at PaDEL website). In the past, it has been shown that all the chemical descriptors do not correlate with the biological activity^33^. So, it is better to remove unnecessary descriptors, which can create noise or over-fitting in the model. To select appropriate set of descriptors for developing QSAR models, first we applied “RemoveUseless” function using Weka software^34^ at default settings (maximum variance percentage, M = 99), that remove descriptors, which either do not vary or vary too much at maximum variance percentage. Second, we applied “CfsSubsetEval” as attribute evaluator with “BestFirst” as search method with default settings in the forward direction (lookup size, D = 1 and amount of backtracking, N = 5) again using Weka software^34^. In the third step, we applied F-stepping^35^ technique to further reduce the non-significant descriptors. In F-stepping, we took all the descriptors obtained from “BestFirst” algorithm together and checked the performance by removing each descriptor one by one (this is also called as backward direction of descriptor selection). If the performance is increased or unaffected by removing a descriptor, we permanently removed that descriptor. On the other hand, if the performance is decreased by removing a descriptor, we put back that descriptor and this cycle was repeated for each descriptor. Finally, we used these minimally selected descriptors as input for training and testing of SVM based QSAR models using leave-one-out cross-validation technique.

#### (iv) G-scales Descriptors

We have adopted G-scales descriptors from the study by Wang *et al.*^17^ and these descriptors were derived from the 457 kinds of physicochemical properties available in AAindex database^36^. Out of this large set of physicochemical properties, only eight properties were selected using stepwise multiple regression (SMR) and used as descriptors to characterize the peptides. We have used these eight descriptors (G1 - G8) for the development of QSAR models because of their superior performance as shown by the Wang *et al.*^17^.

### Support Vector Machine (SVM)

In this study, we have employed a well-known supervised machine learning technique ‘Support Vector Machine’ for developing both regression and classification models^37^. In our study, we have implemented SVM using SVM^*light*^ software (version 6.02), which is freely available at http://www.cs.cornell.edu/People/tj/svm_light/. SVM^*light*^ is a user-friendly software allowing the user to implement various kernels *e.g.* linear, polynomial, radial or sigmoid.

### SVM based Regression Models

For tiny peptides, we have developed regression models using input features like amino acid composition, atomic composition and chemical descriptors. A regression model tries to correlate the input features with the biological activity (pIC_50_) and predicts the biological activity (dependent variable) of unknown peptide on the basis of input features (independent variables). Two major reasons account for developing regression models for tiny peptides. First, classification models were not possible due to limited number of negative AHT peptides and second; we got the pIC_50_ values for sufficient number of AHT peptides to develop the regression-based models. This tiny class contains dipeptides and tripeptides, and we developed separate regression models for both of them.

### SVM based Classification Models

Classification models predict the specific class to which a new peptide belongs (here two classes are AHT and non-AHT) on the basis of learning on training set. For the classification of two classes, SVM tries to draw a hyperplane or a set of hyperplanes separating the two classes by ensuring largest distance with the nearest data-points of two classes. But in most of the cases, two classes are not linearly separable. Therefore, SVM uses kernel functions k(x,y) e.g. polynomial, radial or sigmoid, which is also called as kernel trick. In our study, we used radial basis function (RBF) as kernel option. In SVM^*light*^, one can define the kernel option by setting the value of t (t = 2 for RBF) to apply the appropriate kernel. For small, medium and large AHT peptides, we have developed classification models and we used randomly generated peptides as negative class (non-AHT peptides).

### Evaluation of models

In this paper, we used leave-one-out cross-validation technique (LOOCV) for training and testing our models. LOOCV is a standard method commonly used for evaluating the performance of machine-learning models. For evaluating the performance of our regression models, we calculated the following standard parameters; Pearson’s correlation coefficient (R) and root mean square error (RMSE). For classification models, we have calculated sensitivity, specificity, accuracy and MCC. MCC is considered as the most robust parameter^38^ for evaluating the prediction method. The MCC value ‘1’ corresponds to the perfect prediction, whereas ‘0’ points to a completely random prediction.

## Results

### Analysis of antihypertensive peptides

**Figure 2:**
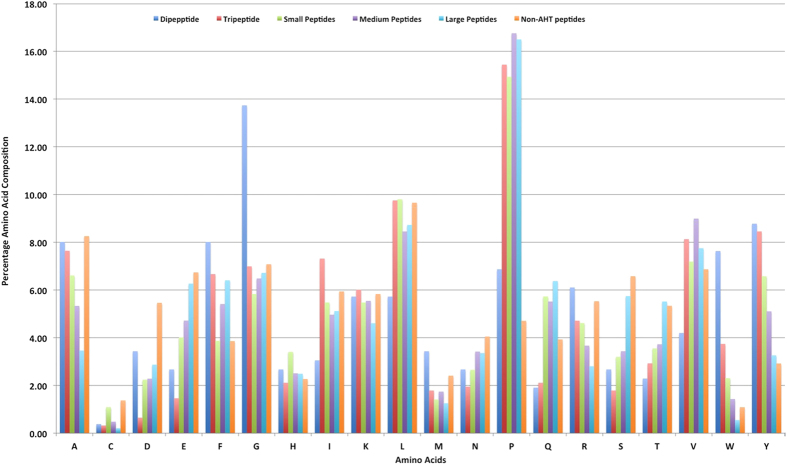
Amino acid composition of different class of AHTs with Non-AHTs.

Generally, antihypertensive peptides may vary from two amino acids to 15 amino acids length^14^. We computed percent amino acid composition of peptides belonging to different categories like tiny, small, medium and large. For understanding the bias in the residue occurrence, we have computed amino acid compositions of non-AHTPs (randomly generated peptides from Swiss-Prot), called reference amino acid composition. As shown in Table 2, Tryptophan and Tyrosine are highly abundant in antihypertensive dipeptides; Phenylalanine and Glycine also frequently occur in AHT dipeptides. In contrast, certain residues (like Cysteine, Glutamic acid, Serine) are not preferred in AHT dipeptides. In the case of AHT tripeptides, Proline, Tryptophan and Tyrosine frequently occur whereas residues like Aspartic Acid, Glutamic acid, Asparagine occur rarely (Fig. 2). Certain types of residues occur frequently in all classes of AHTPs like Proline, which is highly abundant in all types of AHTPs. Similarly, amino acids like Aspartic Acid, Serine is less frequent in most of the AHTPs in comparison to non-AHTPs (Table 2 and Fig. 2).

### Performance of various SVM models

In this study, we built two types of models for antihypertensive peptide prediction, depending upon the type of dataset. First, we have developed regression-based SVM models for tiny peptides *viz*, dipeptides and tripeptides. For small, medium and large peptides, we have developed classification models.

#### (i) Regression-based models for Tiny peptides

Regression-based models have been developed for predicting pIC_50_ values of tiny peptides. Here, we used three types of features i.e. amino acid composition, atomic composition and chemical descriptors to develop models. First, we used amino acid composition and achieved Pearson’s correlation coefficient (R) of 0.605 and 0.218 for dipeptides and tripeptides respectively (Table 3). Next, we used atomic composition and achieved maximum correlation (R) of 0.611 and 0.315 for dipeptides and tripeptides, respectively (Table 3). Then, we used selected PaDEL descriptors of dipeptides (*n* = 12) and tripeptides (*n* = 20) (Supplementary Tables 1 and 2) to develop SVM based models and achieved maximum correlation (R) of 0.701 and 0.543 for di- and tripeptides respectively (Table 3). In case of G-scales descriptors, we achieved correlation of 0.681 and 0.353 for di- and tripeptides respectively. In order to check the predictability of our models, we evaluated them on independent dataset (external validation). Our dipeptide models achieved the maximum, correlation coefficient of 0.762 using atomic composition (Table 3). In case of tripeptides, we achieved maximum correlation coefficient of 0.379 using PaDEL descriptors. Our tripeptide models failed to achieve high performance on independent dataset; it may be due to limited set of data (Table 3).

#### (ii) Classification models for small peptides

In the case of small peptides, we have developed SVM based classification models using amino acid composition, atomic composition and PaDEL descriptors. In case of tetrapeptides, PaDEL descriptors achieved highest accuracy of 76.67% with MCC of 0.53 (Table 4). For pentapeptides, atomic composition performed better and achieved accuracy and MCC of 72.04% and 0.44 respectively (Table 4). Again for hexapeptides, atomic composition achieved highest accuracy of 77.39% with MCC of 0.55 (Table 4).

#### (iii) Classification models for medium peptides

**Figure 3:**
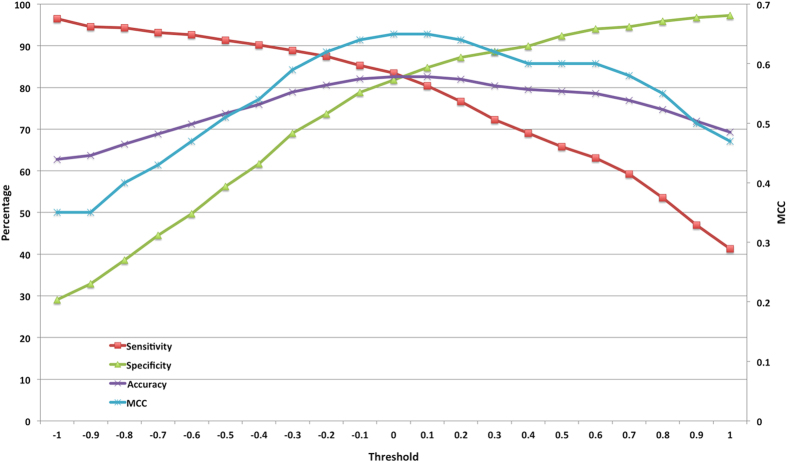
SVM threshold wise performance of medium peptides using amino acid composition.

Generally, atomic composition and chemical descriptors based methods are not reliable for longer peptides (length greater than 6 amino acids). Moreover, calculating chemical descriptors of longer peptides for building models is computer-intensive. So, we developed amino acid composition based and atomic composition based models for these classes of peptides and achieved maximum accuracy of 82.61% and 82.34% respectively with MCC of 0.65 in both the cases (Table 5). Figure 3 illustrates the SVM threshold wise results of amino acid composition for medium peptides.(iv)

#### Classification models for large peptides

**Figure 4:**
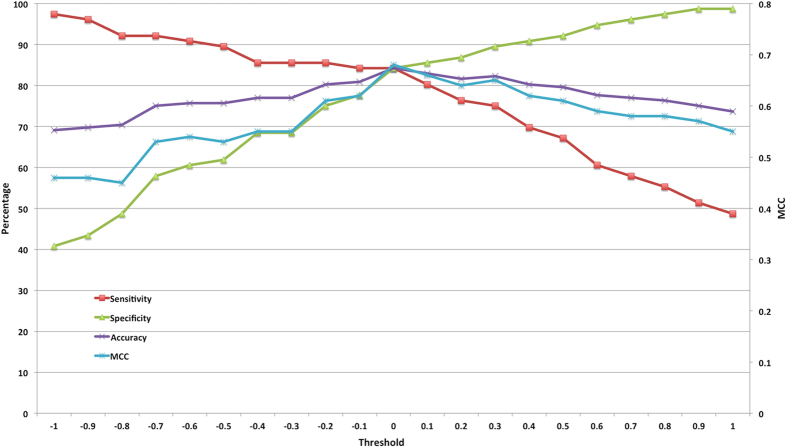
SVM threshold wise performance of large peptides using amino acid composition.

In this case, we have developed models for peptides having length more than 12 residues. For these peptides also, we have developed two kinds of models. First, using amino acid composition, where we have achieved maximum accuracy of 84.21% with MCC of 0.68 (Table 5). Second model was developed using atomic composition, where we have achieved maximum accuracy of 82.24% with MCC of 0.65 (Table 5). Figure 4 illustrates the SVM threshold wise results of amino acid composition for large peptides.

### Comparison with previous methods

It is not possible to compare our models directly with previous methods, as previous methods used small datasets for developing models. Recently, Wang *et al.*^17^ developed models for predicting antihypertensive activity of peptides and demonstrated that their method achieved better performance than previous methods. Thus we trained and tested our models on Wang *et al.*^17^ dataset (58 dipeptides, 55 tripeptides) and achieved highest correlation coefficient (R) 0.851 and 0.857 for di- and tripeptides respectively using atomic composition. The performance of our models was better or comparable with the previous studies^17^. Wang *et al.*^17^ got highest performance using G-scales, we test G-scales on our datasets and observed that our PaDEL descriptors outperformed the G-scales descriptors in case of both di- and tripeptides (Table 3). Moreover, the performance of G-scales descriptors on our datasets was very less as compared to the performance on their own datasets. It seems that G-scales is over optimize for dataset used by Wang *et al.*^17^. On the other hand, our PaDEL based descriptors and atomic composition performed reasonably well on both the datasets, which support a wide applicability domain of our descriptors as compared to the G-scales descriptors. It is not possible to compare our classification models developed on small, medium and large peptides because no such method is reported in literature.

### Web server

With the purpose of providing service to the community, we have developed a web server known as AHTpin (http://crdd.osdd.net/raghava/ahtpin/). This webserver has major modules for designing, screening and mapping of peptides on proteins. Designing peptide module allows the user to generate all possible analogs and predict antihypertensive property of analogs. Screening peptides of AHTpin enables the user to identify antihypertensive peptides from a library of peptides. Server also helps the users to identify regions in a protein that have antihypertensive peptides. AHTpin also provides the physicochemical properties of each processed peptide, which are displayed in a sorting-enabled table.

## Discussion

There are numerous studies that report the occurrence of antihypertensive peptides in various sources *e.g.* wheat, potato, vegetable, meat, egg, etc. Also, a number of synthetic compounds, which act as ACE inhibitors, are already available in the market for the treatment of hypertension. Since, synthetic drugs have numerous side effects; the inclination towards nature-derived or natural antihypertensive molecules is highly desired. There are few QSAR studies, which predicted the antihypertensive nature of small peptides, mainly di- and tripeptides^18^. Most of the previous studies have used smaller datasets and have developed only correlation or QSAR models. The present study has been made to emanate predictive QSAR models in addition to the classification models. These models were developed using the largest available datasets of antihypertensive peptides till date. In our amino acid composition analysis, we have found that glycine is the most prevalent amino acid in AHTs having length of two residues. Interestingly proline was found to be predominantly present in tripeptides, small peptides, medium peptides and large peptides. A scrutiny of different types of features in prediction methods becomes necessary to find the most significant ones associated with prediction. It was noticed that descriptor-based regression model performs better than simple amino acid and atomic composition based model for tiny peptides. Due to the short length, their information is not represented by amino acid composition. Hence, descriptor-based regression model performed best in these cases. In comparison to the previously used descriptors (G-scales), our descriptors performed well on our large datasets, which supports their wide predictability. We developed our models in compliance with the OECD principles^20^, where we achieved reasonable performance in external validation (on independent datasets) to show the robustness and predictability of our models. We have also provided all the information about our models e.g. procedure, input features, datasets etc. on the website, which is also in accordance with OECD. For peptides having length equal or more than four amino acids, we developed classification models. For developing classification models, randomly generated peptides were used as negative datasets in the absence of experimentally validated negative datasets. The ratio of positive and negative datasets is very critical to develop robust machine-learning models. One recent chemoinformatics study by Kurczab *et al*.^39^ showed that the preferable ratio of positive to negative is 1:9 to 1:10. But in a sequence based bioinformatics approach, the ratio of positive to negative datasets should be 1:1 to avoid any imbalance and biased learning^40^,^41^, so we took negative peptides in equal ratio of positive peptides to develop our models. We found that our classification models performed well on all the three features *viz.* amino acid composition. Medium and large peptides have adequate length to represent them by amino acid composition vector. So, amino acids composition performed better in case of medium and large peptide datasets. To help researchers working in this area and scientific community, all these models have been integrated in the form of a web server called “AHTpin”, which is freely available at the URL: http://crdd.osdd.net/raghava/ahtpin.

## Additional Information

**How to cite this article**: Kumar, R. *et al.* An *in silico* platform for predicting, screening and designing of antihypertensive peptides. *Sci. Rep.* **5**, 12512; doi: 10.1038/srep12512 (2015).

## Supplementary Material

Supplementary Information

## Figures and Tables

**Table 1:** Atomic composition of 20 natural amino acids.

Amino Acids	Type of Atoms					Total number of bonds	Number of single bonds	Number of Double bonds
	C	H	N	O	S			
A	3	7	1	2	0	12	11	1
C	3	7	1	2	1	13	12	1
D	4	7	1	4	0	15	13	2
E	5	9	1	4	0	18	16	2
F	9	11	1	2	0	23	19	4
G	2	5	1	2	0	9	8	1
H	6	9	3	2	0	20	17	3
I	6	13	1	2	0	21	20	1
K	6	14	2	2	0	23	22	1
L	6	13	1	2	0	21	20	1
M	5	11	1	2	1	19	18	1
N	4	8	2	3	0	16	14	2
P	5	9	1	2	0	17	16	1
Q	5	10	2	3	0	19	17	2
R	6	14	4	2	0	25	23	2
S	3	7	1	3	0	13	12	1
T	4	9	1	3	0	16	15	1
V	5	11	1	2	0	18	17	1
W	11	12	2	2	0	28	23	5
Y	9	11	1	3	0	24	20	4

**Table 2:** Amino acid composition of different types of AHTPs and non-AHTPs.

Residue	Dipeptide	Tripeptide	Small peptide	Medium peptide	Large peptide	Non-AHT
A	8.02	7.64	6.61	5.33	3.46	8.26
C	0.38	0.33	1.09	0.48	0.21	1.37
D	3.44	0.65	2.24	2.29	2.87	5.46
E	2.67	1.46	4.01	4.72	6.27	6.74
F	8.02	6.67	3.87	5.41	6.41	3.86
G	**13.74**	6.99	5.83	6.49	6.72	7.08
H	2.67	2.11	3.41	2.51	2.49	2.27
I	3.05	7.32	5.48	4.97	5.12	5.94
K	5.73	6.02	5.47	5.55	4.61	5.83
L	5.73	9.76	9.80	8.45	8.73	9.66
M	3.44	1.79	1.41	1.75	1.26	2.41
N	2.67	1.95	2.66	3.42	3.36	4.05
P	6.87	**15.45**	**14.93**	**16.76**	**16.50**	4.71
Q	1.91	2.11	5.73	5.52	6.38	3.93
R	6.11	4.72	4.62	3.67	2.80	5.53
S	2.67	1.79	3.20	3.44	5.75	6.58
T	2.29	2.93	3.55	3.73	5.52	5.34
V	4.20	8.13	7.19	8.99	7.75	6.87
W	7.63	3.74	2.31	1.43	0.55	1.09
Y	8.78	8.46	6.58	5.11	3.26	2.92

Amino acids with significantly high composition are shown in bold.

**Table 3:** The Performance of SVM based regression models on leave-one-out cross-validation and external validation.

Peptide Class	Features	Cross Validation		External Validation	
		R	RMSE	R	RMSE
Dipeptides	Amino acid	0.605	0.978	0.759	1.047
	Atomic	0.611	0.936	0.762	0.972
	Descriptors	0.701	0.830	0.663	0.998
	G-scales	0.681	0.848	0.669	0.977
Tripeptides	Amino acid	0.218	0.995	0.285	1.151
	Atomic	0.315	1.009	0.189	1.383
	Descriptors	0.543	0.821	0.379	0.999
	G-scales	0.353	0.988	0.029	

*R: Pearson correlation coefficient; **RMSE:** Root Mean Square Error.

**Table 4:** The performance of classification models on small peptides.

Peptides Class	Features	Sensitivity	Specificity	Accuracy	MCC
Tetrapeptide	Amino Acid	71.24	79.74	75.49	0.51
	Atomic	70.59	79.74	75.16	0.51
	Descriptors	76.67	76.67	76.67	0.53
Pentapeptide	Amino Acid	70.74	70.37	70.56	0.41
	Atomic	72.96	71.11	72.04	0.44
	Descriptors	71.11	63.70	67.41	0.35
Hexapeptide	Amino Acid	72.36	80.90	76.63	0.53
	Atomic	74.87	79.90	77.39	0.55
	Descriptors	81.91	70.5	76.19	0.53

*MCC: Matthews-correlation coefficient.

**Table 5:** The performance of classification models on medium and large peptides.

Peptides Class	Features	Sensitivity	Specificity	Accuracy	MCC
Medium Peptides	Amino Acid	83.42	81.79	82.61	0.65
	Atomic	81.25	83.42	82.34	0.65
Large Peptides	Amino Acid	84.21	84.21	84.21	0.68
	Atomic	84.21	80.26	82.24	0.65

*MCC: Matthews correlation coefficient.
