# Supplementary material for: An in silico platform for predicting, screening and designing of antihypertensive peptides
Source: Sci Rep. 2015 Jul 27;5:12512. doi: 10.1038/srep12512 (PMC4515604; doi:10.1038/srep12512)
Supplement: Supplementary Information [file srep12512-s1.doc]

**An *in silico* platform for predicting, screening and designing of antihypertensive peptides**

Ravi Kumar^#^, Kumardeep Chaudhary^#^, Jagat Singh Chauhan^#^, Gandharva Nagpal^#^, Rahul Kumar^#^, Minakshi Sharma, Gajendra P.S. Raghava^*^

Bioinformatics Centre, CSIR-Institute of Microbial Technology, Chandigarh-160036, India.

^*^ Corresponding author

**^#^**Authors contributed equally

**Email: raghava@imtech.res.in**

Bioinformatics Centre, CSIR-Institute of Microbial Technology, Chandigarh-160036, India

Phone: +91-172-2690557

Fax: +91-172-2690632

**Table 1: Descriptors used for the development of dipeptide QSAR models.**

|  | **Descriptors** | **Class** | **Description** |
| --- | --- | --- | --- |
| **1** | ATSm4 | Auto correlation Descriptor Mass | ATS autocorrelation descriptor, weighted by scaled atomic mass  (2D) |
| **2** | nHBint7 | Electrotopological State Atom Type Descriptor | Count of E-State descriptors of strength for potential Hydrogen Bonds of path length 7 (2D) |
| **3** | nsOm | Electrotopological State Atom Type Descriptor | Count of atom-type E-State: -O- (2D) |
| **4** | minHBd | Electrotopological State Atom Type Descriptor | Minimum E-States for (strong) Hydrogen Bond donors (2D) |
| **5** | PetitjeanNumber | Petitjean Number Descriptor | Petitjean number (2D) |
| **6** | XLogP | XLogP Descriptor | XLog P (2D) |
| **7** | FP186 | CDK Fingerprint | Fingerprint of length 1024 and search depth of 8 |
| **8** | FP509 | CDK Fingerprint | Fingerprint of length 1024 and search depth of 8 |
| **9** | FP728 | CDK Fingerprint | Fingerprint of length 1024 and search depth of 8 |
| **10** | MACCSFP128 | MACCS fingerprint | ('*~[CH2]~*~*~*~[CH2]~*',0), # ACH2AAACH2A |
| **11** | PubchemFP690 | Pubchem Fingerprint | O-C-C-C-C-C-O |
| **12** | PubchemFP699 | Pubchem Fingerprint | O-C-C-C-C-C(C)-C |

**Table 2: Descriptors used for the development of tripeptide QSAR models.**

|  | **Descriptors** | **Class** | **Description** |
| --- | --- | --- | --- |
| **1** | ETA_BetaP_s | Extended Topochemical Atom Descriptor | A measure of electronegative atom count of the molecule relative to molecular size (2D) |
| **2** | ExtFP58 | Extended Fingerprint | Extends the Fingerprinter with additional bits describing ring features |
| **3** | ExtFP751 | Extended Fingerprint | Extends the Fingerprinter with additional bits describing ring features |
| **4** | GraphFP173 | CDK graph only fingerprint | Specialized version of the Fingerprinter which does not take bond orders into account |
| **5** | GraphFP353 | CDK graph only fingerprint | Specialized version of the Fingerprinter which does not take bond orders into account |
| **6** | GraphFP598 | CDK graph only fingerprint | Specialized version of the Fingerprinter which does not take bond orders into account |
| **7** | GraphFP833 | CDK graph only fingerprint | Specialized version of the Fingerprinter which does not take bond orders into account |
| **8** | MACCSFP115 | MACCS fingerprint | ('[CH3]~*~[CH2]~*',0), # CH3ACH2A |
| **9** | PubchemFP17 | Pubchem Fingerprint | >= 8 N |
| **10** | PubchemFP639 | Pubchem Fingerprint | O-C-C-C-O |
| **11** | SubFP169 | Substructure Fingerprint | Presence of SMARTS Patterns for Functional Group Classification by Christian Laggner |
| **12** | KRFP697 | Klekota-Roth Fingerprint | Presence of chemical substructures |
| **13** | KRFP840 | Klekota-Roth Fingerprint | Presence of chemical substructures |
| **14** | KRFP842 | Klekota-Roth Fingerprint | Presence of chemical substructures |
| **15** | KRFP4125 | Klekota-Roth Fingerprint | Presence of chemical substructures |
| **16** | KRFP4225 | Klekota-Roth Fingerprint | Presence of chemical substructures |
| **17** | SubFPC100 | Substructure Fingerprint Count | Count of SMARTS Patterns for Functional Group Classification by Christian Laggner |
| **18** | KRFPC483 | KlekotaRoth Fingerprint Count | Count of chemical substructures |
| **19** | KRFPC3946 | KlekotaRoth Fingerprint Count | Count of chemical substructures |
| **20** | KRFPC4125 | KlekotaRoth Fingerprint Count | Count of chemical substructures |
